# Lipid Modifications in Cilia Biology

**DOI:** 10.3390/jcm8070921

**Published:** 2019-06-27

**Authors:** Kasturi Roy, Ethan P. Marin

**Affiliations:** Department of Internal Medicine, Section of Nephrology, Yale School of Medicine, PO Box 208029, New Haven, CT 06520-8029, USA

**Keywords:** palmitoylation, myristoylation, prenylation, acylation, ciliopathies

## Abstract

Cilia are specialized cellular structures with distinctive roles in various signaling cascades. Ciliary proteins need to be trafficked to the cilium to function properly; however, it is not completely understood how these proteins are delivered to their final localization. In this review, we will focus on how different lipid modifications are important in ciliary protein trafficking and, consequently, regulation of signaling pathways. Lipid modifications can play a variety of roles, including tethering proteins to the membrane, aiding trafficking through facilitating interactions with transporter proteins, and regulating protein stability and abundance. Future studies focusing on the role of lipid modifications of ciliary proteins will help our understanding of how cilia maintain specific protein pools strictly connected to their functions.

## 1. Background

The primary cilium is an organelle that protrudes from the surface of a cell and functions as a signaling hub. It is a solitary, non-motile, polarized structure serving as a sensory organelle for the cell. It is found on almost every cell type in vertebrates and has been linked to a variety of human diseases, collectively referred to as ciliopathies.

The output of the genetic blueprint of the human body is far more complex and varied than the genes themselves. Protein function is modulated and diversified by post-translational modifications (PTMs), such as phosphorylation, glycosylation, ubiquitination, nitrosylation, methylation, acetylation and lipidation [1]. These covalent alterations of amino-acid side chains have a role in all the aspects of cell biology. Thus, understanding PTMs is important for the understanding and prevention of human diseases.

The structural integrity and function of cilia requires a specific array of proteins. Cilia maintain a specific protein signature by selective trafficking of proteins to and from the cilia. However, how proteins travel to the cilia is still being explored. In this review, we will focus on how lipid modifications are involved in the trafficking of specific ciliary proteins and in the regulation of signaling pathways. Lipid modifications may help proteins travel to the cilia by two (possibly overlapping) mechanisms: (a) Facilitate binding of cilia-targeted proteins to membranes to allow for their delivery to the cilium, or (b) facilitate interactions with specific chaperones that deliver proteins to the cilium.

Proper protein trafficking to and from the cilium is a requisite that ensures normal functioning of ciliary signaling cascades. The primary cilium is reported to have a high density of receptors, transporters and ion channels that play an important role in sensing extracellular stimuli, such as chemicals, light, osmolarity, temperature, and gravity [2]. Cilia provide a separate cellular compartment for the temporal and spatial regulation of signaling molecules, despite being continuous with the cytosol. Primary cilia are critical for a number of signaling pathways: Wnt signaling, calcium signaling, growth factor signaling, Shh signaling, G-protein-coupled receptor (GPCR) signaling and receptor tyrosine kinase signaling [2,3],. Accurate localization of signaling proteins confers proper functioning of the pathways.

As no study to date has shown that proteins are produced in the cilium, the specificity in the ciliary protein array is regulated by protein traffic. The trafficking of proteins to the cilia is complex and several mechanisms have been put forward to explain it. A periciliary diffusion barrier separates the ciliary and plasma membrane despite the continuity between the two [4]. Besides lateral transport along the membrane, specific traffic at the base of the cilium is believed to occur. At the base of the cilium there is a transition zone (TZ) which regulates the entry of proteins such as ADP ribosylation factor-like protein 13B (Arl13b), inositol polyphosphate-5-phosphatase E (INPP5E), adenylate cyclase 3 (ADCY3) and Smoothened (Smo) [5]. A TZ complex of Meckel syndrome (MKS) and Joubert syndrome (JS) proteins regulates the protein transport to the cilia [6,7]. Mutations in the components of the complex compromise TZ function and, in turn, cilia protein composition. The organization and composition of the TZ is still under investigation, but it is plausible that structural components in the region create a lipid microdomain, partitioning the ciliary domain from the extraciliary region. Lipidated proteins pass this region with the help of components like UNC119, PDE6δ and Arl3, which are discussed in later sections. It has been proposed that, like the nuclear pore, a ciliary pore exists in this transition zone, controlling the entry of proteins into the cilia [8]. This hypothesis is consistent with previous research, which has reported that ciliary entry of a cytoplasmic protein, the kinesin-2 motor KIF17 and the X-linked retinitis pigmentosa protein (RP2) require an import signal similar to a nuclear localization signal (NLS) [9,10]. The small GTPase Ran and its binding partners (the importins) have been implicated in regulating cilia entry of specific proteins [11,12]. Bardet–Biedl syndrome (BBS) proteins form a conserved eight subunit protein complex called the BBSome, which particularly localize to the basal body of cilia and centrosomes [13]. It has been proposed that the BBSome forms a protein coat on vesicles, helping the transport of proteins to the cilium [14]. However, recent studies have shown that the BBSome is a cargo adaptor for cilia-bound proteins that are unable to directly bind to intraflagellar transport proteins (IFTs) [15]. Protein trafficking is also reported to be facilitated by the presence of specific cilia-targeting sequences (CTSs). However, there is no unique CTS, suggesting more than one molecular mechanism is involved. Some of the best characterized CTSs are the amino acid sequences RVxP and VxPx, which exist on polycystin 2 (PC2) and rhodopsin, respectively [11,16]. The Ax[S/A]xQ motif has been reported to occur on several GPCRs, which localize to the primary cilium [17]. Studies have proposed that the targeting of G-protein-coupled receptors may involve multiple and even competitive mechanisms. Exploring these mechanisms is important, though care should be taken regarding quantification methods of protein enrichment in cilia considering its specific geometry [18]. Apart from the CTSs, lipid modifications have been identified as a requirement for the trafficking of several ciliary proteins, a few of which will be discussed here. About 30% of cilia proteins are likely to be acylated [19]. With more protein acylation studies being carried out, it is becoming clear that acylation of ciliary proteins allows for more than just membrane attachment. Along with membrane tethering, lipid modifications are also integral for proper localization, distribution, abundance, stability and function of several cilia proteins [19,20,21,22,23,24]. The role of various lipid modifications (Table 1) on cilia protein trafficking and signaling will be discussed in the following sections (Table 2).

## 2. Palmitoylation

Palmitoylation has a huge impact on cellular functions, with 10% of the proteome palmitoylated, but is nevertheless understudied compared to other PTMs. Palmitoylation is a lipidation process in which a C16 palmitoyl group from palmitoyl-coenzyme A (CoA) is added to the thiolate side chain of cysteine residues via palmitoyl acyl transferases (PATs) (Table 1) [54]. Palmitoylation is unique because of its reversible nature and the great number of transferases and thioesterases involved in the process. This modification can anchor proteins to the membrane and thioesterases can break the link between the protein and the lipid, making it an on/off switch for regulation of membrane localization [55,56]. The reversibility of palmitoylation makes it a candidate regulator for localization and function of many proteins. Several ciliary proteins are known to be palmitoylated, some of which we will discuss here. It is interesting to note that none of the PATs have been found to localize in the cilium, indicating that it is not the site of the modification [19].

Several proteins require attachment of a palmitoyl group for proper localization, which in turn affects its abundance and function. Ciliary proteins are no different. Fibrocystin is the gene product of the *PKHD1* gene and localizes to the primary cilia. Mutations in this gene cause autosomal recessive polycystic kidney disease (ARPKD). The CTS of fibrocystin has three palmitoylated cysteine residues [31,57]. The targeting sequence alone—without palmitoylation—is not enough for ciliary targeting in non-transmembrane fusion proteins. However, recent studies with transmembrane domains like native proteins have shown that palmitoylation of the CTS is not necessary [58]. This makes it reasonable to believe that palmitoylation may help in restricting the protein to specific lipid domains and help in recruitment of sorting complexes for delivery to the cilia.

Arl13b is a GTPase localized primarily in the cilia and is necessary for proper cilia formation. Palmitoylation of mouse Arl13b at cysteines 8 and 9 in its N-terminal region has been shown to be essential for its localization to the cilia, its stability, and its function in cilia elongation and formation [19]. Though palmitoylation is absolutely necessary for the cilia elongation function, Arl13b localization to the cilia also requires an intact C-terminus [19,33].

Many GPCRs are palmitoylated at the C-terminus region. Rhodopsin is a seven-transmembrane GPCR and is the primary photoreceptor molecule of vision. It was the first GPCR reported to be palmitoylated [59,60]. The palmitates (at cysteines 322 and 323) anchor the C-terminus to the membrane, thus forming a fourth intracellular loop/domain. Rhodopsin is synthesized and folded in the endoplasmic reticulum and further processed in the Golgi. From there it is delivered to the primary cilium and the cilium-derived sensory organelles—the rod outer segments (ROS) [34]. It has two cilia-targeting signals, VxPx and the FR [34]; these motifs are recognized by Arf4 and ASAP1, respectively. FR motif mutants of rhodopsin (FR-AA) fail to interact with ASAP1 and are therefore unable to localize to the cilia in IMCD3 cells. The role of palmitoylation of rhodopsin remains unclear, but it is thought to orient/compartmentalize the protein near the membrane in such a manner so as to provide maximum interaction with components, facilitating its trafficking and function [35]. Palmitoylation-deficient rhodopsin reportedly localizes normally to the outer segment of intact rods (ROS) in mice. Further, palmitoylation—but not localization—of rhodopsin helps to prolong its activity by attenuating phosphorylation by rhodopsin kinase [59]. A lack of palmitoylation destabilizes the molecular interactions that occur in the C-terminal region and hinders the activation of transducin [61,62]. Recent studies report that palmitoylation of rhodopsin is important for oligomerization-dependent raftophilicity (the affinity of membrane proteins to the lipid raft [63]) and thus its compartmentalization [64]. Palmitoylation has also been reported to play a role in the stability of opsin: An unliganded form of rhodopsin. A lack of opsin palmitoylation causes its mislocalization and rapid rod photoreceptor degeneration [37,65].

*PKD1* and *PKD2* encode the polytopic integral membrane proteins polycystin 1 (PC1) and polycystin 2 (PC2), which localize to the primary cilia. Mutations in either PC1 or PC2 are at the core of autosomal dominant polycystic kidney disease (ADPKD). It is a genetic renal condition, characterized by numerous renal cysts, which progressively leads to loss of renal function. Recently, we reported that PC1, but not PC2, is palmitoylated [66]. Data indicate that palmitoylation is important for its localization and abundance.

A very recent study by Wu et al. from Harvard Medical School showed that the transcription factor regulatory factor X 3 (RFX3) is S-fatty acylated on a cysteine residue, which is evolutionarily conserved in the dimerization domain. RFX3 is one of the key transcription factors involved in cilia formation and function. The acylation of RFX3 regulates the protein interaction network by affecting homo- or hetero-dimerization, thereby regulating ciliogenesis and tissue specification. Deregulation of fatty acid metabolism may affect fatty acylation of RFX3 and thereby the RFX3-mediated protein interaction network, leading to ciliopathies and metabolic disorders such as diabetes [39,40].

Hedgehog signaling is an evolutionarily conserved pathway, having an important role in the development of numerous organs and cancer [67]. Lipid modifications on the hedgehog (Hh) protein regulate its function and distribution. It has palmitate in an amide linkage with N-terminal cysteine and there is a cholesterol moiety addition on the C-terminus. The acylation efficiency and specificity depend in part upon prior cholesterol modification [68]. Dual modifications provide higher membrane affinity of Hh, thus allowing its association with sterol-rich membrane microdomains in *Drosophila*, and to lipid rafts in mammalian cells. Such associations provide a platform for intracellular sorting, signal transduction, and restriction of its range of activity. However, membrane affinity is not the only function of the lipidation process, as removal of N-terminal cysteine—and, hence, acylation—results in a large reduction in signaling function [41]. The N-terminus may be required for modulating additional interactions, which are themselves needed for the complete signaling process. Pepinsky’s group from Biogen, Inc., Cambridge, proposed that the N-terminal region of Hh could be important for modulating the patched 1/smoothened (Ptc-1/Smo) interaction, and that its truncated forms are unable to trigger the de-repression of Smo despite being able to bind Ptc-1 [69,70].

Another important signaling pathway associated with cilia is the Wnt pathway. The Wnt protein is palmitoylated, giving the protein its hydrophobic character and is important for its extracellular transport. Murine Wnt-3a is also modified with a monounsaturated fatty acid—palmitoleic acid—at a conserved serine residue (Ser209) [71]. The loss of palmitate through its enzymatic removal or a cysteine mutation has been shown to reduce the biological activity of Wnt, indicating that lipid modification is integral to the signaling pathway [42,70].

## 3. N-Myristoylation

N-myristoylation is the covalent attachment of a 14-carbon saturated fatty acid, myristate, to the N-terminal glycine of proteins by N-myristoyltransferase (Table 1) [26]. It can occur on a variety of proteins with diverse function and localization. It is an irreversible protein modification that generally occurs co-translationally following the removal of the initiator methionine residue by methionylaminopeptidases. Myristoylation can also occur post-translationally, as in the case of many apoptotic proteins, wherein proteolytic cleavage by caspases reveal an internal myristoylation motif [23]. Myristoylation has been reported to help a number of ciliary proteins with membrane association required for cilia localization, but it is not sufficient for ciliary localization. The presence of a second binding partner or a chaperone protein in the process is required.

Myristoylation, along with the presence of specific CTSs, has been implicated in the trafficking of many ciliary proteins. Cystin is a 145 amino acid (aa) cilium-associated protein responsible for autosomal recessive polycystic kidney disease (PKD). Like other cystoproteins (e.g., polycystin 1), its expression is developmentally regulated. Cystin is myristoylated at its glycine 2 residue and this modification helps in its membrane association. It has also been shown that this acylation is required for its proper localization to the ciliary membrane [27]. However, acylation is not sufficient for its targeting to the cilium. Cystin has a unique AxEGG motif necessary for its targeting and retention in the cilium.

Cilia localization of another protein, nephronophthisis 3 (NPHP 3), requires a coiled-coil (CC) domain and myristoylation at the N-terminus. Nephronophthisis is an autosomal recessive kidney disease with 11 causative genes identified up to date (*NPHP 1*–*11*). Mutations in NPHP 3 cause human NPHP type 3. The mouse *NPHP3* gene product is a 140 kDa protein (1325 amino acids) with multiple domains, including three coiled-coil (CC) domains and eight tetratricopeptide repeats (TPRs). The CC domain is required to transport NPHP 3 to the basal body and myristoylation is required for its subsequent transport into the ciliary shaft. Secondary binding partners may interact with the CC or the myristoylated region to facilitate the process, but that mechanism is still unknown [43]. NPHP 3 has also been implicated in both canonical and noncanonical Wnt signaling [72,73]. Hence, proper lipid modification of the protein and its accurate localization to the cilia is important for ciliary signaling.

**Figure 1:**
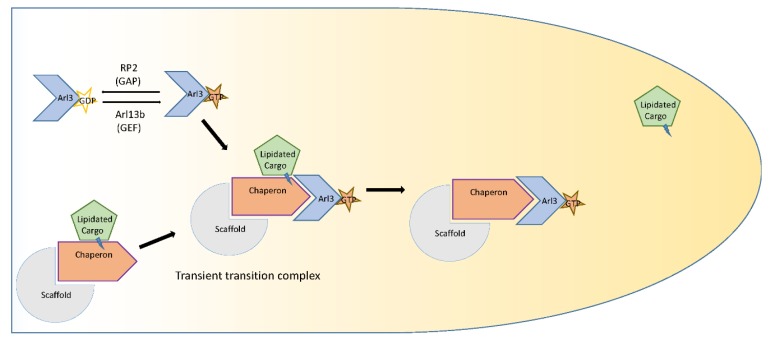
Schematic representation of lipidated (myristoylated/prenylated) protein delivery to the cilium.

An interesting mode of chaperone-mediated, myristoylated/prenylated protein delivery to the cilia has been recently identified. Two novel proteins, UNC119A and UNC119B, have been shown to act as chaperones. These novel proteins have specificity for a diverse subset of myristoylated proteins, such as G-protein α subunits [74], NPHP 3 [75] and Src-type tyrosine kinase and deliver them to the cilium. UNC119 polypeptides bind lauroyl (C12) and myristoyl (C14) side chains via a hydrophobic pocket, formed by an immunoglobulin-like β-sandwich fold. Once the cargo-bound chaperones reach the cilium, Arl3-GTP releases the cargo from the chaperone by allosteric displacement, delivering the proteins to the cilium [75,76,77,78]. The targeting of NPHP 3 to the cilia requires Arl3, UNC119B (not UNC119A) and the Arl3 GAP RP2. Knockdown of the Arl3 system reduces the percentage of cilia with localized NPHP 3 [75]. This is a sophisticated delivery system, wherein the lipids do not merely act as a membrane tethering chain, but as a specific binding site for protein–protein interactions (Figure 1).

Cilia both release and bind extracellular vesicles (EVs). Myristoylation has been implicated in the signaling and targeting of proteins to EVs in Jurkat T cells [79]. CIL-7 is a ciliary protein known to regulate EV biogenesis and is required for polycystin-mediated sensory signaling. Recent studies in *Caenorhabditis elegans* (*C. elegans*) have indicated that myristoylation is important for CIL-7 function and its targeting to EVs [44].

## 4. Double Acylation: Myristoylation and Palmitoylation

Non-receptor tyrosine kinases, G-protein subunits and G-protein regulators show more than one lipid modification, which modulate their localization and function [24,28]. Some ciliary proteins also have more than one acylation, and this modification is required for correct membrane and subcellular localization. Myristoylation and palmitoylation on proteins for membrane anchoring and ciliary localization is seen in eukaryotic flagella, *C. elegans* sensory neurons, mammalian photoreceptors and retinal pigment epithelial cells [35,45,80].

Though many cilia proteins are double acylated, which helps in strong membrane association, in many cases, it is not the only purpose of the lipid molecules. The presence of double acylation on various proteins may confirm specific binding partners or localization for specific functions. *C. elegans*’ protein phosphatase with EF-hands (CePPEF), the only PPEF family of serine/threonine protein phosphatases encoded within *C. elegans,* needs adjacent N-terminal myristoylation and palmitoylation for proper cilia trafficking. As palmitoylation is sufficient for membrane binding, the functional consequence of having both modifications is unclear. Ramulu and Nathans, of the Johns Hopkins University School of Medicine, provide an explanation that in *C. elegans*, palmitoylation of CePPEF may be heterogeneous, such that one subset of the CePPEF proteins is palmitoylated and strongly membrane-associated, while a second subset of the CePPEF proteins carries only a myristate group and can therefore shuttle between the membrane and the cytosol [45].

The flagellar protein calflagin, or its homolog flagellar calcium-binding protein (FCaBP), is both myristoylated and palmitoylated, and these modifications are necessary for its flagella localization. It is a unique protein that uses a calcium–acyl switch for regulated membrane attachment and probable association with interactors similar to the protein recoverin. Recoverin is a neuronal calcium sensor, mainly expressed in retinal photoreceptors [81,82]. It is myristoylated in the calcium-bound state and associates with the plasma membrane via interactors. When calcium levels drop, the myristoyl group becomes sequestered in a hydrophobic cleft, leading to loss of membrane association. The calcium-binding state of recoverin regulates the membrane accessibility of its fatty acid, which in turn modulates the binding of recoverin to its partner proteins [20,83,84,85]. Like recoverin, the association of the myristoyl group with the plasma membrane and an association with a partner protein through palmitate are predicted to result in the localization of FCaBP to the flagella [83].

For RP2, lipidation does more than just membrane association. RP2 is mutated in X-linked retinitis pigmentosa. It localizes to the ciliary base and associates with the membrane by dual acylation of the N-terminus via myristoylation at glycine 2 and palmitoylation at cysteine 3 [46]. RP2 traffics to the cilia by interacting with the protein importin, and membrane association of RP2 is a prerequisite for formation of an RP2–importin-β2 complex [10]. A recent study by Yoshimura et al. at Kyoto University, Japan, has shown that, similar to the molecular targeting signal for motile flagella, a combination of myristoylation and palmitoylation targets proteins such as Lck, Fyn and Yes-1 to the primary cilium in eukaryotic cells. The targeting is mediated by an N-terminal dual lipidation-coupled ciliary targeting signal (nlCTS). Palmitoylation of nlCTSs appears to be important not only for ciliary targeting, but also for the stability of the respective proteins [21].

There are reports that proteins which depend on BBSomes for their export from cilia are double acylated; dual lipid modifications are a prerequisite for their accumulation in cilia and they depend on the BBSome system for their exit from there [15]. In *Chlamydomonas reinhardtii*, mutations in the BBSome cause accumulation of proteins—phospholipase D (PLD) and STPK, an AMP-regulated kinase (AMPK)—in the cilia. Both proteins are predicted to have dual fatty acid modifications at the N-terminus [86]. Membrane anchoring by acylation is required—but not sufficient for—their transport by the IFT/BBS system. The loss of BBSome-dependent exclusion of the proteins leads to their trapping, and hence their accumulation in the cilia.

## 5. Prenylation: Farnesylation and Geranylgeranylation

Prenylation is a lipid modification wherein a farnesyl (15-carbon) or geranylgeranyl (20-carbon) isoprenoid is covalently added to the cysteine residue of the CaaX box at or near the C-terminus of proteins (Table 1) [30]. Apart from facilitating membrane attachment, prenylation is also known to be involved in protein–protein interactions, influencing protein function and localization. It is in fact believed that lipid association is just a part of the process by which prenylated proteins associate with membranes. They generally interact with receptor molecules in the membranes [22,30,87]. As recent studies support this theory, this indicates that in some cases protein prenylation is important for facilitating interaction with specific receptor modules, rather than just membrane tethering.

A few ciliary proteins are known to be prenylated, wherein proper localization of these proteins and their functions may be attributed to their lipid attachment. Many inherited retinal diseases have been associated with mutations causing prenylation defects [29].

Inositol polyphosphate-5-phosphatase B (INPP5B) is an important ciliary protein, the knockdown of which leads to cilia defects in zebra fish. For proper cilia localization, it needs to be prenylated at its C-terminus [47]. Deletion of the CaaX amino acid motif leads to abolition of INPP5B recruitment to the cilia and also reduces cilia length.

Studies from the last few years have provided interesting evidence that prenylated proteins are trafficked to the cilia with the help of the chaperone protein phosphodiesterase 6δ (PDE6δ), which is similar to the previously discussed case of myristoylated proteins [88,89]. PDEδ is known to be involved in the outer segment (modified cilium) targeting of PDE6 α/β subunits, the transducin γ subunit, and GRK1 in photoreceptors. The proteins are either partly mis-localized or are degraded in PDEδ-null photoreceptors [89]. However, our understanding of the mechanistic aspect of the role of PDEδ in trafficking of proteins is limited. Rheb is a small G-protein involved in regulating mTORC1 (the mammalian target of rapamycin complex 1). Rheb gets farnesylated at its C-terminal CaaX box. Recently, Wittinghofer et al. from the Max Planck Institute showed that the lipid moiety of farnesylated Rheb is deeply buried in a hydrophobic pocket of PDEδ, indicating that binding occurs through a prenyl group in the C-terminus [88]. Thus, the lipid here is a site of interaction and not just a modification for membrane tethering. Retinitis pigmentosa GTPase regulator (RPGR) is an interacting partner for PDEδ proteins. It is a prenylated protein that acts as GTP–GDP exchange factor for Rab8, a protein important for ciliary protein transport. Geranylgeranylation is necessary for the correct localization of RPGR in both the Golgi apparatus and cilia [49,90]. RPGR is prenylated at the C-terminus and mutation of the CaaX prenylation motif abrogates the localization [90]. Structural studies suggest that RPGR helps to recruit prenylated ciliary cargo proteins on PDEδ at the ciliary transition zone. The cargo is delivered to the cilia, where they are allosterically displaced from the chaperone by Arl3 (Figure 1), similar to the myristoylated cargos mentioned earlier [88,91]. Arl13b acts as a guanine nucleotide exchange factor (GEF) for Arl3 and activates Arl3 in the cilia, which releases the cargo from the carrier protein [92]. However, the release mechanism of myristoylated cargo from UNC119A is different from that of prenylated cargo release from PDEδ, in that Arl3 binding to PDEδ leads to a closed (rather than an open) conformation, restricting cargo binding [76].

INPP5E is also a ciliary protein and is involved in the ciliopathies Joubert syndrome (JS) and MORM syndrome (an autosomal recessive congenital disorder). Unlike INPP5B, INPP5E has a CTS (FDRELYL) along with a CaaX domain, and the CTS is necessary but not sufficient for its ciliary localization [50]. The CaaX motif reportedly has a role in MORM syndrome, wherein a mutation causes the premature truncation of the INPP5E protein, thereby causing the protein to lose its C-terminal CaaX domain [93]. INPP5E associates with PDE6δ to traffic to the basal body. Some studies have reported that, in hTERT–RPE1 cells, INPP5E requires Arl13b but not Arl3 or Arl2 for its unloading from PDE6δ and cilia entry [50]. Specifically, INPP5E interacts with both Arl13b and PDE6δ individually, but Arl13b and PDE6δ never interact physically [50,51]. This indicates that Arl13b facilitates ciliary targeting of INPP5E by directly interacting with INPP5E and promoting its release from PDE6δ, unlike the ARL3–PDE6δ interaction [50]. Recent clinical reports, however, have shown that Arl3 is also required for INPP5E unloading in cilia. This indicates that ciliary proteins form different hubs that cross talk and interact together [52,94]. Such structural studies have strengthened the role of the lipid as more than just a membrane binding moiety. Though new models are being proposed [50,76,88], detailed mechanisms for cilia trafficking of several prenylated proteins, such as PDE6 α/β and GNGT1, remain to be determined.

## 6. Conclusion and Future Questions

Cilia are a specialized cellular structure characterized by a specific protein signature. Much attention has been paid to understand how proteins get sorted to the cilium, where they ultimately exert their precise function. More than one transport mechanism is involved in the process. The targeting of proteins to the cilia depends on the recruitment of specific sorting complexes and also on the correct partitioning into lipid micro-domains. A great number of studies have shown that lipid modifications of proteins play an important role in trafficking, as well as in the biology of the cilium. Lipid modifications have a far greater role than just tethering the proteins to the membrane. Though a number of ciliary proteins are acylated, a complete understanding of the role of lipids in protein transport, function and localization in cilia is still at a nascent stage. New studies focusing on the delivery of lipidated cargo to cilia are being pursued, but more needs to be done. For instance, it will be interesting to test if lipid modifications act as signals for ciliary protein localization, degradation and more. Additionally, identification of interactors, using various biochemical and structural studies of the acylated cargos, will open up new mechanistic pathways. Also, the site of ciliary protein lipidation is not fully known. Detailed studies on protein modifications and their role in cilia are needed to broaden our understanding of primary cilia and ciliopathies.

## Figures and Tables

**Table 1:** Common lipid modifications at a glance.

	Palmitoylation	N-Myristoylation	Prenylation
**Structure**	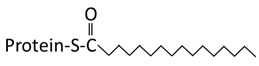 Saturated 16 carbon palmitate addition to cysteines on the targeted proteins via the formation of a thioester linkage	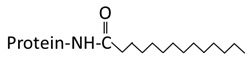 Saturated 14 carbon myristoyl group addition by an amide bond to the α-amino group of an N-terminal glycine	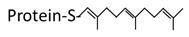 Transfer of either a farnesyl or a geranyl- geranyl moiety to the cysteine of the CaaX motif of the target protein
**Reversibility**	Reversible	Irreversible	Irreversible
**Enzymes**	A number of enzymes involved in the process: 24 Palmitoyltransferase (also known as PATs or DHHCs) and ~15 acyl protein thioesterase (APT)	N-myristoyltransferase	Farnesyltransferase, Geranylgeranyltransferase I, Geranylgeranyltransferase II
**Motif**	Can occur on cysteines located at different sites in a protein	N-myristoylation occurs at the N-terminus at MGxxxS motif	Occurs only at the CaaX boxes at the C-terminus
**Example**	eNOS, Arl13b [19,25]	Src, Cystin [26,27]	G-protein γ subunit, AIPL1 [28,29,30]

**Table 2:** List of lipid-modified ciliary and flagellar proteins discussed in this review.

Protein	Site of Lipid Modification	Lipid Modification Identification Method	Construct Used to Identify Lipid Modification	Cilia Targeting Requirements (CTS and More)	Disease Association/Function	Reference
Fibrocystin	Palmitoylation on three conserved cysteine residues in CTS	Mutation and metabolic labeling	193 residues of C-terminal	18 intracellular aa flanking the transmembrane domain, including conserved cysteines	ARPKD	[31,32]
Arl13b	Palmitoylation in N-terminal region	Mutation and metabolic labeling	Full length protein	Multiple regions in the protein	JS	[19,33]
Rhodopsin	Palmitoylation in C-terminal cytoplasmic region	Enzymatic and chemical cleavage techniques, tandem mass spectrometry, mutations/deletions and knock-out mice	C-terminal fusion protein	VxPx and the FR (in IMCD3)	Inherited retinal degenerative diseases	[34,35,36]
PC1	Palmitoylation in C-terminal region	Metabolic labeling and Biochemical assays	~200-amino-acid C-terminal tail (CTT)	Multiple sites in the coiled-coil motif in the C-terminal tail including VxP motif and multiple cis-acting elements	Mutation cause ADPKD	[37,38]
RFX3	Palmitoylation on a cysteine residue in the dimerization domain	Biochemical assays and mass spectrometry	Full length protein	-	Ciliopathies and metabolic disorders, like diabetes	[39,40]
Hedgehog	Palmitoylation in N-terminal cysteine	Mass spectrometry and metabolic labeling	Full length protein	-	Organ development and cancer	[41]
Wnt	Palmitoylation on a conserved cysteine in N-terminal region	Mass spectrometry, biochemical, enzymatic methods and mutations	Full length protein	-	Wnt signaling involved in animal development including proliferation of stem cells	[42]
Cystin	Myristoylation on glycine 2	Mutations and metabolic labeling	Full length and various truncated mutants	AxEGG	PKD	[27]
NPHP3	Myristoylation at the N-terminus	Metabolic labeling and mutations	Truncated N-terminal fusion proteins	N-terminal CC domain and the myristoylation site	Nephronophthisis	[43]
CIL-7	Myristoylation at the N-terminus	Mutations	Full length protein	Myristoylation motif	PKD	[44]
CePPEF	N-terminal myristoylation and palmitoylation	Metabolic labeling and mutations	Full length protein and N-terminal recombinants	N-terminal region, palmitoylation is particularly important	Calcium regulation	[45]
Calflagin	Myristoylation at glycine 2 and palmitoylation at cysteine 3 in the N-terminal region	Biochemical assays, metabolic labeling and mutations	Full length protein	Palmitoylation	Calcium binding protein	[20]
RP2	Myristoylation at glycine 2 and palmitoylation at cysteine 3 in the N-terminal region	Mutation	Truncated N-terminal fusion proteins	N-terminal dual lipidation-coupled ciliary targeting signal (nlCTS)	X-linked retinitis pigmentosa	[21,46]
INPP5B	Prenylated at its C-terminus CaaX	Mutation and knock downs	Full length protein	Prenylation	Important for retrograde trafficking	[47,48]
RPGR	Prenylated at its C-terminus CaaX	Mutation	Full length and deletion mutants	Two independent ciliary targeting signals: one within the RLD and the other near the C-terminus.	Inherited retinal degenerative diseases	[49]
INPP5E	prenylated at its C-terminus CaaX	Biochemical assays, enzymatic assays and mutations	Full length protein	FDRELYL (not sufficient, require other interactors)	JS and MORM syndrome	[50,51,52,53]

## Abbreviations

ADCY3	Adenylate cyclase 3
ADPKD	Autosomal dominant polycystic kidney disease
AIPL1	Aryl-hydrocarbon-interacting protein-like 1
AMPK	AMP-regulated kinase
APT	Acyl protein thioesterase
Arl2	ADP ribosylation factor-like protein 2
Arl3	ADP ribosylation factor-like protein 3
Arl13b	ADP ribosylation factor-like protein 13B
Arf4	ADP ribosylation factor 4
ASAP1	ArfGAP with SH3 domain, ankyrin repeat and PH domain 1
BBS	Bardet–Biedl syndrome
CC	Coiled-coil
CePPEF	*Caenorhabditis elegans* protein phosphatase with EF-hands
CIL-7	A ciliary protein
CTS	Cilia targeting sequence
eNOS	Endothelial nitric oxide synthase
EV	Extracellular vesicles
FCaBP	Flagellar calcium-binding protein
GAP	GTPase activating protein
GEF	Guanine nucleotide exchange factor
GNGT1	Guanine nucleotide-binding protein G(T) subunit gamma-T1
GPCR	G-protein-coupled receptors
GRK1	G-protein-coupled receptor kinase 1
GTP	Guanosine triphosphate
GTPase	Guanosine triphosphatase
Hh	Hedgehog
hTERT RPE-1	hTERT-immortalized retinal pigment epithelial cell line, hTERT RPE-1, was derived by transfecting the RPE-340 cell line with the pGRN145 hTERT-expressing plasmid
IFT	Intraflagellar transport
IMCD3	Inner medullary collecting duct3
INPP5E	Inositol polyphosphate-5-phosphatase E
INPP5B	Inositol polyphosphate-5-phosphatase B
JS	Joubert syndrome
KIF17	Kinesin family member 17
mTORC1	Mammalian target of rapamycin complex 1
MKS	Meckel syndrome
nlCTS	N-terminal dual lipidation-coupled ciliary targeting signal
NPHP	Nephronophthisis
PATs	Palmitoyl acyl transferases
PC1	Polycystin 1
PC2	Polycystin 2
PDE6δ	Phosphodiesterase 6δ
PLD	Phospholipase D
PKD	Polycystic kidney disease
*PKHD1*	Human autosomal recessive polycystic kidney disease gene
PPEF	Protein phosphatases with EF-hand domains
Ptc-1	Patched 1
PTM	Post-translational modifications
REP-1	Rab escort protein-1
RLD	RCC1-like domain
RFX3	Regulatory factor X3
RP2	Retinitis pigmentosa protein
RPGR	Retinitis pigmentosa GTPase regulator
TPRs	Tetratricopeptide repeats
TZ	Transition zone
Smo	Smoothened
STPK	Serine/threonine protein kinase
